# Uncertainty in the response of terrestrial carbon sink to environmental drivers undermines carbon-climate feedback predictions

**DOI:** 10.1038/s41598-017-03818-2

**Published:** 2017-07-06

**Authors:** D. N. Huntzinger, A. M. Michalak, C. Schwalm, P. Ciais, A. W. King, Y. Fang, K. Schaefer, Y. Wei, R. B. Cook, J. B. Fisher, D. Hayes, M. Huang, A. Ito, A. K. Jain, H. Lei, C. Lu, F. Maignan, J. Mao, N. Parazoo, S. Peng, B. Poulter, D. Ricciuto, X. Shi, H. Tian, W. Wang, N. Zeng, F. Zhao

**Affiliations:** 1 School of Earth Sciences and Environmental Sustainability, Northern Arizona University, P.O. Box 5694, Flagstaff, Arizona 86011-5694 USA; 2 Department of Global Ecology, Carnegie Institution for Science, Stanford, California USA; 3 Woods Hole Research Center, Falmouth, MA 02540 USA; 4 Laboratoire des Sciences du Climat et de l’Environnement, IPSL-LSCE CEA CNRS UVSQ, 91191 Gif sur, Yvette France; 5 Environmental Sciences Division and Climate Change Science Institute, Oak Ridge National Laboratory, Oak Ridge, TN 37831 USA; 6 National Snow and Ice Data Center, Cooperative Institute for Research in Environmental Sciences, University of Colorado, Boulder, Colorado USA; 7 Jet Propulsion Laboratory, California Institute of Technology, Pasadena, CA USA; 8 School of Forest Resources, University of Maine, Orno, ME USA; 9 Atmospheric and Global Change Division, Pacific Northwest National Laboratory, Richland, WA USA; 10 National Institute for Environmental Studies, Tsukuba, Japan; 11 Department of Atmospheric Sciences, University of Illinois at Urbana-Champaign, Urbana, IL USA; 12 Atmospheric Sciences and Global Change Division, Pacific Northwest National Laboratory, Richland, Washington USA; 13 State Key Laboratory of Hydroscience and Engineering, Department of Hydraulic Engineering, Tsinghua University, Beijing, China; 14 Department of Ecology, Evolution and Organismal Biology, Iowa State University, Ames, IA USA; 15 Department of Ecology, Montana State University, Bozeman, MT USA; 16 International Center for Climate and Global Change Research and School of Forestry and Wildlife Sciences, Auburn University, Auburn, Alabama USA; 17 Ames Research Center, National Aeronautics and Space Administration, Moffett Field, California USA; 18 Department of Atmospheric and Oceanic Science, University of Maryland, College Park, Maryland USA

## Abstract

Terrestrial ecosystems play a vital role in regulating the accumulation of carbon (C) in the atmosphere. Understanding the factors controlling land C uptake is critical for reducing uncertainties in projections of future climate. The relative importance of changing climate, rising atmospheric CO_2_, and other factors, however, remains unclear despite decades of research. Here, we use an ensemble of land models to show that models disagree on the primary driver of cumulative C uptake for 85% of vegetated land area. Disagreement is largest in model sensitivity to rising atmospheric CO_2_ which shows almost twice the variability in cumulative land uptake since 1901 (1 s.d. of 212.8 PgC vs. 138.5 PgC, respectively). We find that variability in CO_2_ and temperature sensitivity is attributable, in part, to their compensatory effects on C uptake, whereby comparable estimates of C uptake can arise by invoking different sensitivities to key environmental conditions. Conversely, divergent estimates of C uptake can occur despite being based on the same environmental sensitivities. Together, these findings imply an important limitation to the predictability of C cycling and climate under unprecedented environmental conditions. We suggest that the carbon modeling community prioritize a probabilistic multi-model approach to generate more robust C cycle projections.

## Introduction

The terrestrial carbon (C) cycle plays a critical role in regulating the accumulation of C in the atmosphere^1, 2^ and recent work suggests that the strength of the terrestrial sink is growing^1–4^. Some^3, 5^ argue that CO_2_ fertilization is the predominant driver of the growth in the terrestrial C sink, particularly in the tropics. However, others suggest that models may be overly sensitive to changes in atmospheric CO_2_ concentration^6^, calling into question our understanding of C cycle-climate feedbacks. Beyond CO_2_ fertilization, other factors such as nitrogen (N) deposition^7^, forest regrowth^8^, high latitude warming^9^, and an increase in growing season length^3^ can all contribute to an increased terrestrial sink, while drought^10^, conversion of forests to agriculture^11^, and emerging N^12, 13^ and phosphorus (P)^13, 14^ limitations can act to constrain terrestrial C uptake. The balance of these interacting factors is complex and unknown, yet determines whether the terrestrial biosphere will serve as a net source or sink of C to the atmosphere. For example, while CO_2_ fertilization may be a dominant driver for the *influx* of C to the biosphere, its importance for the *net* land C sink, relative to the full suite of environmental factors, is unclear. The ambiguity in attributing changes in global land C uptake to key drivers is, in part, due to the difficulty of extrapolating locally observed relationships to global scales. The use of models to quantify the sensitivity of land C uptake to changing biophysical (i.e. climate) and biogeochemical (e.g., land-cover change history, CO_2_ concentration, N deposition) factors, although having great potential, has been limited with many recent studies focusing on individual factors^5^, specific regions^15^, and/or only one or two models^16, 17^.

Here we use an ensemble of twelve models from the Multi-scale Synthesis and Terrestrial Model Intercomparison Project (MsTMIP)^18^ and a series of sensitivity simulations (refer to Methods) to attribute changes in historical global land C uptake (and loss) to key biophysical and biogeochemical drivers. Because models vary widely in their representation of land-atmosphere C dynamics^19^, each model can be viewed as one possible realization of terrestrial C cycling and its key drivers. Furthermore, because MsTMIP models are run using a common protocol — forcing data, steady-state spin up, and sensitivity simulations are uniform across all models^18, 20^ — differences in predictions reflect differences in model process representations^19^. This makes it possible to quantify the contribution of biophysical and biogeochemical drivers to changes in the terrestrial C cycle and to assess the influence of model structure. Variability in model sensitivities to environmental change speaks to the potential reliability of future projections of terrestrial C-cycle behavior and feedbacks with climate.

## Results and Discussion

**Figure 1:**
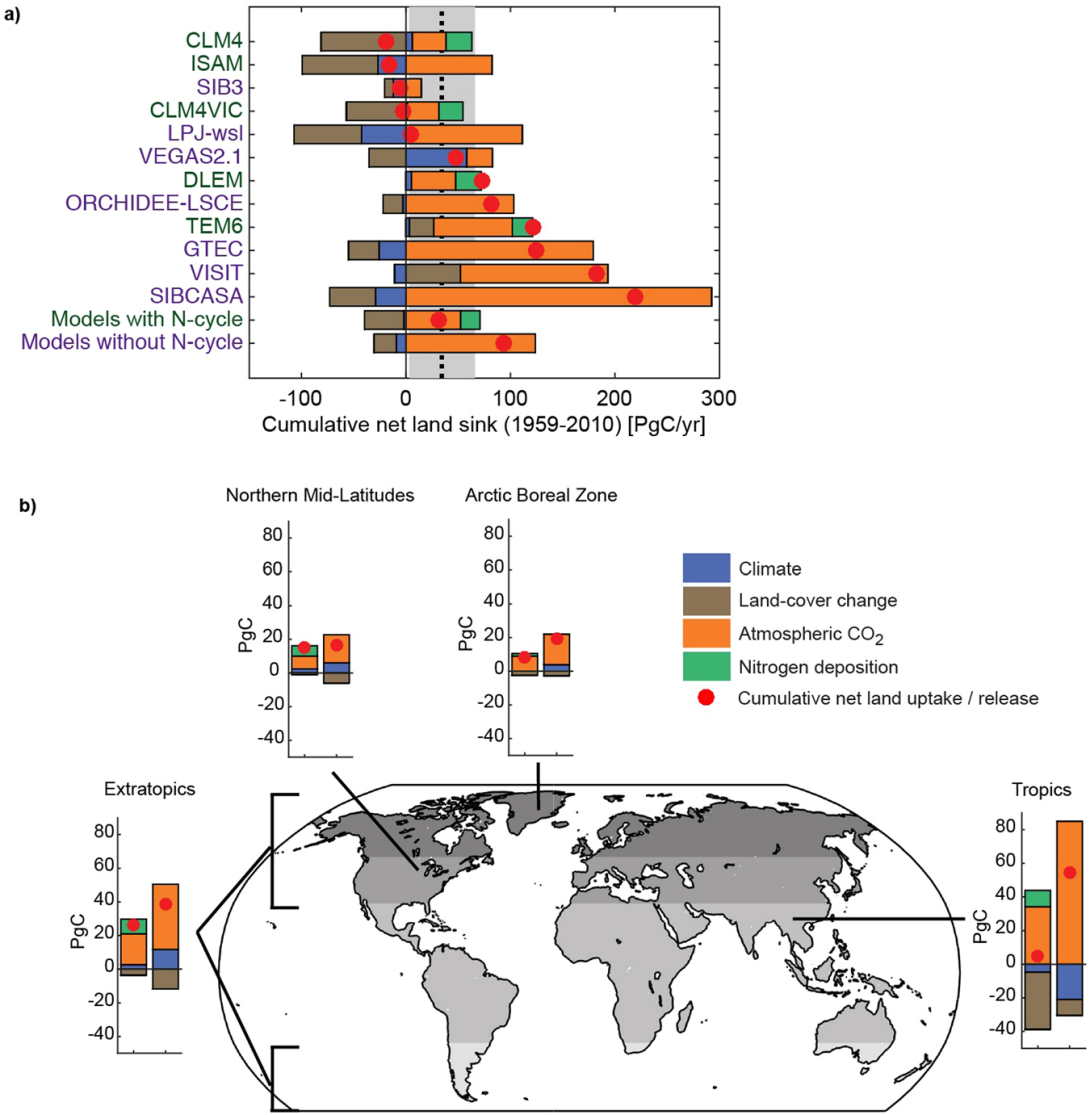
Attribution of net global carbon land sink. (**a**) Attribution of the cumulative net land sink (red circles) by model and for the two sub-ensemble means (models with and without a coupled carbon-nitrogen cycle) from 1959 to 2010 compared to the cumulative sink (black dashed line) and associated uncertainty (grey shaded region) estimated from the Global Carbon Project^6^. The cumulative sink is decomposed into the influence of time varying climate (blue), land-cover change history (brown), atmospheric CO_2_ (orange), and nitrogen deposition (green). (**b**) Attribution of the cumulative net land carbon sink (red circles) over period 1959 to 2010 by region for ensemble mean of models with (left) and without (right) a coupled carbon-nitrogen cycle. Negative values indicate carbon loss from the terrestrial biosphere, where positive values indicate a carbon gain by the terrestrial biosphere. Figure was created using Matlab version R2015a (http://www.mathworks.com/products/matlab/) with post processing done in Adobe Illustrator CS6 Version 16.04 (https://www.adobe.com/products/illustrator.html).

We find that models disagree on both the magnitude of CO_2_ fertilization and the relative importance of other environmental drivers to cumulative land C uptake (Fig. 1a). The strength of the CO_2_ fertilization effect is highly variable (mean ± s.d.: 94.1 ± 80.6 PgC cumulative uptake since 1959; a time period consistent with global C cycle estimates from the Global Carbon Project (GCP)^2^), and other factors have an equal if not greater influence on cumulative land C uptake/release in many models (Table S1). For example, the impact of historical land cover change (LCC) (−28.0 ± 40.1 PgC cumulative C loss since 1959; Fig. 1a; Table S1) is second to that of CO_2_ fertilization, with high variability across models in the tropics (−19.7 ± 31.7 PgC cumulative C loss since 1959; Table S2). While the size of C stocks is an important factor in determining future carbon storage and loss^21^, we find no significant relationship between initial pool size and cumulative land uptake attributed to any of the key environmental drivers examined (Figure S2). This holds across the simulated range for both steady-state tropical above-ground live biomass (~230 to ~610 PgC) and soil carbon (~210 to ~840 PgC)

Variations in model structure lead to a 200 PgC difference in estimates of the cumulative net land sink since 1959 (Fig. 1a; Table S2). Whether a model considers C and N coupling appears to strongly influence net sink sensitivity to key environmental drivers. On average, the inclusion of a coupled C-N cycle leads to a CO_2_ fertilization response, in terms of cumulative C uptake, that is over 50% weaker relative to C-only models (Fig. 1; Table S1). C-N models also exhibit a dampened sensitivity to historical climate change, with all but one C-N model showing small cumulative C gains, globally, in response to warming (Figure S1). As a result of these more modest sensitivities to CO_2_ and climate, and a large variability in land C response to land-cover change history (LCC; Table S1), models with a coupled C-N cycle estimate a cumulative net sink (31.2 ± 62.6 PgC since 1959) that is a third of that estimated by models without N coupling (93.3 PgC ± 84.1 PgC). This weaker simulated net uptake is more consistent with global mass balance constraints on net biome productivity from the Global Carbon Project^2^ (34.6 ± 31.4 PgC, Fig. 1a; Table S2) inferred from atmospheric CO_2_ observations (Methods), and suggests that C-only models may be missing a key constraint on CO_2_ fertilization, and thus land carbon uptake^7, 21, 22^. The greatest difference in the magnitude of CO_2_ fertilization between C-N and C-only models occurs in the tropics (Fig. 1b; Table S3), where P, not N, is thought to limit plant productivity^21, 23^. The CO_2_ sensitivity of the C-N models is more consistent across models than that of C-only models. It is difficult to say whether this difference in CO_2_ sensitivity in the tropics is due to unrealistic constraints placed on CO_2_ fertilization by nitrogen limitation or other model structural differences^24^. It is possible that the difference between C-only and C-N models is due to an unrealistically strong unconstrained tropical CO_2_ fertilization response in some C-only models (refer to Figure S1). It is also possible that some C-N models underrepresent N availability in the tropics, perhaps as a surrogate for other limitations (i.e., P), not currently accounted for in models^25^.

**Figure 2:**
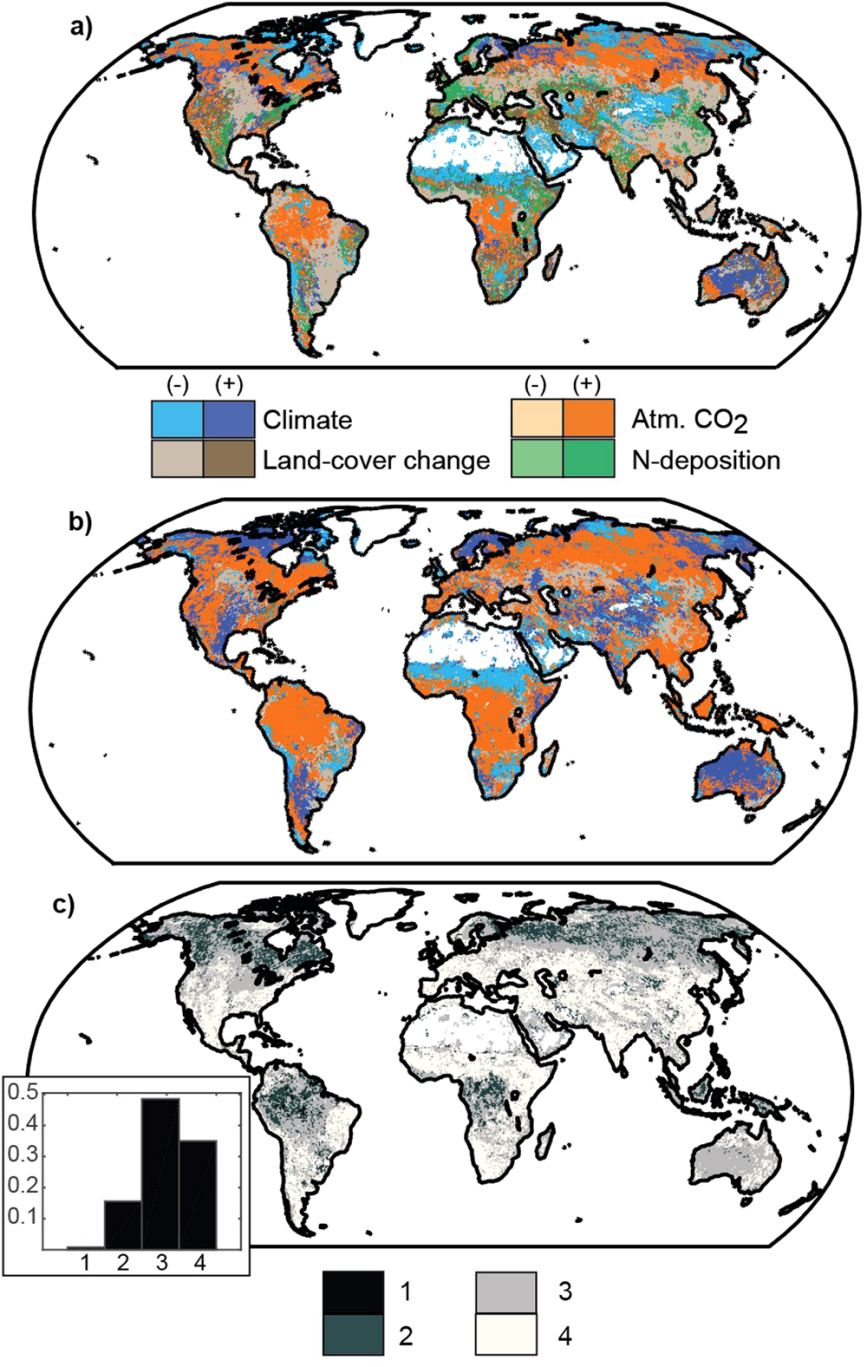
Dominant drivers of cumulative net land sink. (**a**,**b**) Dominant driver of the cumulative net land sink over the simulation period 1959 to 2010 that appears most frequently (i.e., greatest mode) across models (**a**) with (n = 5) and (**b**) without (n = 7) a coupled carbon-nitrogen cycle, and whether that driver is associated with an enhancement (+) or weakening (−) of land carbon sink strength. Drivers include: climate (blue), land cover change history (brown), atmospheric CO_2_ (orange), and N deposition (green). (**c**) The number of different factors (climate, LCC, CO_2_, and N-deposition) identified as the primary driver of cumulative net sink across the full ensemble. Areas with a larger number of possible primary drivers indicate regions with greater disagreement among models. Insets show histograms of fraction of land cells having 1 to 4 different possible drivers. Figure was created using Matlab version R2015a (http://www.mathworks.com/products/matlab/) with post processing done in Adobe Illustrator CS6 Version 16.04 (https://www.adobe.com/products/illustrator.html).

Spatially (Fig. 2a,b; Tables S3–S5), models disagree on the primary driver of cumulative C uptake for over 85% of vegetated land area, where at least three drivers were identified as dominant by different models (Fig. 2c and inset; Figure S3). For example, CO_2_ fertilization is the primary driver of cumulative uptake for anywhere from 11% to 77% of global vegetated land area, depending on the model (Table S6). The inferred importance (or dominance) of an environmental driver (particularly CO_2_) on cumulative C uptake since 1959 is partially explained by whether models include a coupled C-N cycle (Fig. 2a,b). Nevertheless, there is significant disagreement on the spatial importance of all environmental drivers across the full ensemble, particularly in the temperate zone (Fig. 2c; Figure S3). As such, even models with similar estimates of the global cumulative net land sink (e.g., GTEC vs. TEM6 and CLM4 vs. ISAM; Table S2), have widely different sensitivities to key environmental drivers (Figure S1). Despite these large differences, models tend to agree on the relative importance (although not magnitude) of CO_2_ fertilization and climate in parts of the tropics and arctic-boreal zone (Fig. 2c). These are regions that have been identified as potential tipping elements in the coupled C-climate system^26^. Thus, accurately simulating C cycle sensitivity to global change in these regions is key for predicting future climate.

**Figure 3:**
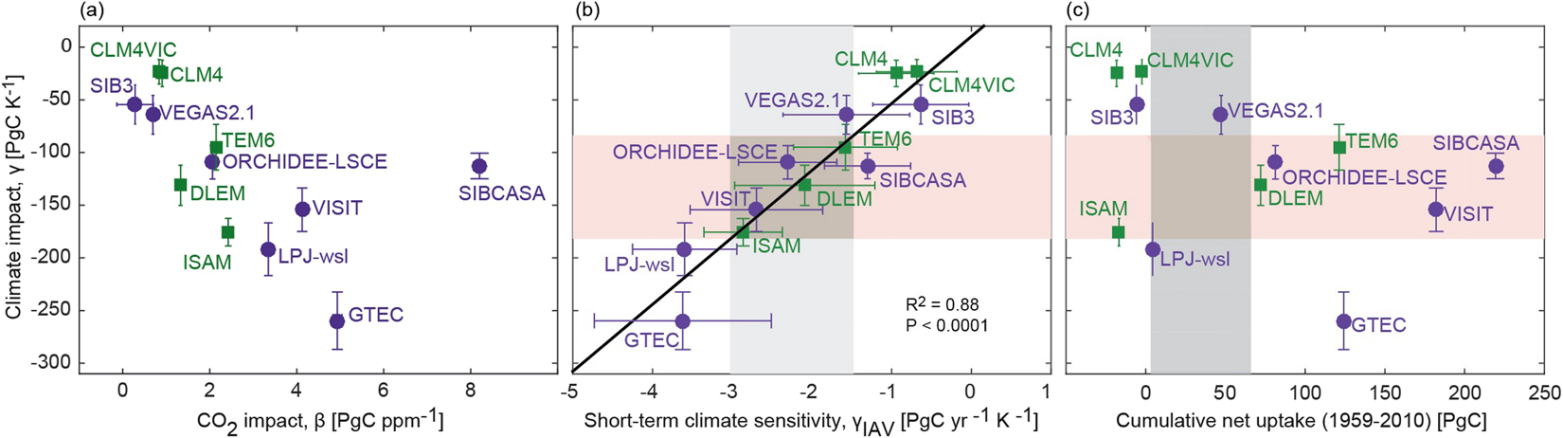
Sensitivity of net land carbon sink to climate and CO_2_. (**a**) The response of the global net land sink over the period 1901 to 2010 to both rising atmospheric CO_2_ (β) and temperature (γ) estimated from linear regression. (**b**) The long-term sensitivity (1901–2010) of the global net land sink to climate warming (γ) versus the short-term sensitivity (1959–2010) of global net uptake to interannual variability in temperature (γ_IAV_). (**c**) The long-term sensitivity of the global net land sink to climate warming (γ) versus global cumulative net uptake of carbon from 1959 to 2010. The error bars show uncertainty in the regression coefficients (β, γ, γ_IAV_) for models with (green) and without (purple) a dynamic nitrogen cycle. The black line in (**b**) shows the best-fit, linear relationship between short- and long-term sensitivities. The vertical light grey shaded region in (**b**) shows the mass-balance constraint (mean ± s.e.) from the Global Carbon Project (GCP) on γ_IAV._ The horizontal red shaded region (in both **b** and **a**) show the constraint on γ_IAV_ (grey shaded region in 3b) mapped onto γ using the model derived relationships (black line in **b**). The GCP mass-balance constraint (mean ± s.d.; from Fig. 1a) on cumulative net uptake is shown as the darker grey shaded region in (**c**). Figure was created using Matlab version R2015a (http://www.mathworks.com/products/matlab/) with post processing done in Adobe Illustrator CS6 Version 16.04 (https://www.adobe.com/products/illustrator.html).

We find that the variability in the importance of key controls results in part from a trade-off between sensitivity to increases in atmospheric CO_2_ (β) and sensitivity to warming (γ) (ρ = −0.89; Fig. 3a) (Methods). In fact, variability in carbon gain attributable to net sink sensitivity to rising atmospheric CO_2_ concentrations (1 s.d. of 212.8 PgC; Table S7) across models is almost double the variability in estimates of the total cumulative land uptake since 1901 (1 s.d. of 138.5 PgC; Table S2; Methods). This tradeoff in CO_2_ – climate sensitivity is strongest in the tropics (Figure S4) and suggests a balancing of model properties (i.e., indirect tuning). Model formulation and parameterization inherently involve making a series of choices that influence model behavior^27, 28^. Some changes are desirable (e.g., model convergence to large-scale constraints), while others may be side effects of the choices made during model development (e.g., implicit compensation of CO_2_ and climate sensitivity). The CO_2_ and climate sensitivities shown here are comparable to the C-concentration and C-climate feedbacks derived from coupled C-climate models^29, 30^ and are not directly tunable; they are instead emergent properties that arise from parameter and other adjustments. A particular historical global land uptake estimate can be obtained using dramatically different assumptions about CO_2_ fertilization and temperature sensitivity, which correspond to different representations of biophysics and biogeochemistry. For example, GTEC and TEM6 diverge by ca. 200% in their sensitivities (Fig. 3a) but have nearly identical cumulative uptake values (Table S2). Although these two models agree for the historical period, they would respond very differently under future environmental conditions, where their modeled response to changes in atmospheric CO_2_ and temperature will differ.

Although observational constraints can, in principle, be used to set bounds on sensitivities and identify models with more plausible representations of biophysical and biogeochemical controls, available data products are insufficient to resolve the difference observed across the MsTMIP ensemble. Recognizing this, previous studies^31, 32^ have used contemporary temperature sensitivities in the tropics to derive constraints on feedbacks in the real climate system for which there are no direct measurements. We use an analogous approach here (Methods), leveraging the mass-balance constraint on net C uptake from GCP^2^ to identify a global observational constraint on the long-term net sink sensitivity to climate of −135 ± 56 PgC K^−1^ (Fig. 3b). We find that none of the models in the ensemble, however, are consistent (within 1 sigma or 68% confidence) with both this derived constraint on climate sensitivity and the magnitude of the GCP constraint on the cumulative global net land sink (Fig. 3c). Our findings demonstrate that models consistent with global constraints are arriving at a “right” answer, but perhaps for the “wrong” reason. Here we find that some models project cumulative land sinks that are consistent with observation-based constraints (e.g., VEGAS2.1 and LPJ-wsl in Fig. 3c), but based on dichotomous sensitivities to climate and CO_2_ concentrations (Fig. 3a), and climate sensitivities that are inconsistent with observationally derived constraints (red shading in Fig. 3b). Conversely, other models exhibit climate sensitivities consistent with observationally-derived constraints (e.g., DLEM, ORCHIDEE-LSCE, and TEM6 in Fig. 3b), but simulate a cumulative global net sink inconsistent with the observationally-based constraints (Fig. 3c).

The trade-off in climate and CO_2_ sensitivity observed here (Fig. 3a), along with uncertainty in model response to key drivers, underscores a key weakness in future climate projections, but also an opportunity for model development and improvement. The indirect tuning implied by this trade-off is systematic of both social anchoring (i.e., once aware of a benchmark typically used for model evaluation, this knowledge invariably influences decisions in model development)^27, 33^ and equifinality (i.e., multiple equality valid parameter sets giving rise to same sensitivities)^34^. This is in contrast to explicit tuning, such as arbitrary adjustments to achieve a more realistic model output^35^. Improvements in terrestrial C cycle (and thus climate) predictability require that models not only produce the right end points (e.g., land sink strength), but also the correct pathways to those endpoints (i.e., sensitivities and the model parameterizations/structure that gives rise to C cycle sensitivities). Identifying and correcting any compensating errors, while far difficult to accomplish, is critical for projecting future climate behavior under altered, even unprecedented, combinations of environmental conditions.

The fact that models disagree significantly on not only the magnitude of the net land sink, but also its sensitivity to changing environmental conditions offers insight into advancing the science and directing the modeling community. While individual models have provided major insight into terrestrial biogeochemistry^9, 36, 37^, the lack of consensus among models is important and should raise a warning flag for modelers and the scientific community. We should be very cautious about over-interpreting the results of a single model, when in fact there is significant breadth in potential responses, and a dearth of observations that can truly validate which response is “correct”. Each model represents a potential pathway in land C cycle response to a changing world. Thus, while each model is useful, embracing an ensemble approach, akin to weather prediction, provides an opportunity for more comprehensive exploration of possible responses.

Probabilistic forecasts are routinely used in climate and weather forecasting, where multi-model ensembles are used to capture and represent uncertainty and inform the likelihood of future predictions^38, 39^; uncertainty that, based on current knowledge and computing power, is unavoidable. In contrast, the numerous carbon cycle model ensembles used to provide insight into terrestrial carbon dynamics^40^ are not routinely used in a such a probabilistic framework. There are both opportunities and challenges in using this type of probabilistic forecast approach to better represent the uncertainty of land carbon uptake predictions under future environmental conditions.

A key challenge centers on robust observations at scales consistent with models, i.e., observations that can truly constrain model sensitivities to changing environmental conditions. The current lack of observational data products, or conversely, multiple yet divergent data products, has made “ground truthing” models a very pliable exercise^33^. Neither a single model, nor a single data product will be sufficient to realistically predict how the carbon cycle will respond to an unprecedented climate future. Within the context of these challenges, the use of probabilistic ensembles allows projections to better reflect the true uncertainty in modeling the terrestrial carbon cycle, including uncertainty in model structure, driver data and initial conditions (e.g., carbon pools size).

Here, using the MsTMIP ensemble of models has made it possible to challenge conclusions from previous studies about the main drivers of the cumulative land sink, by showing that models disagree on the primary driver of cumulative C uptake for 85% of vegetated land area. The use of a relatively large model ensemble also made it possible to demonstrate that across-model variability in the CO_2_ and temperature sensitivity is partly due to indirect model tuning, whereby comparable estimates of C uptake can arise despite a broad range of sensitivities to key environmental conditions. Together, these findings highlight uncertainties in system response to changing environmental conditions, uncertainties that can only be characterized through a multi-model approach.

## Methods

### Model ensemble

The ensemble used here is part of the Multi-scale synthesis and Terrestrial Model Intercomparison Project (MsTMIP)^18^. The models are forced with a consistent set of environmental driver data (Table S8) and a standard spin-up and simulation protocol^18, 20^ over a 110-year period spanning 1901 to 2010. The collection of 12 models samples the structural diversity in current land surface models; many of the models are the land surface modules of climate and Earth System Models, but here and as part of MsTMIP they are run in uncoupled mode with externally defined climate and atmospheric forcings. The use of consistent driver data across all models removes one source of uncertainty and isolates the impact of model structure on model estimates. The protocol^18^ calls for a suite of sensitivity simulations (Table 1), adding one time-varying driver at a time, to quantitatively attribute trends in each model’s estimate of the net land sink to the influence of four key forcing factors: climate, land cover change history, atmospheric CO_2_ concentration, and nitrogen deposition. The impact of each forcing factor is calculated through simulation differencing. We also track how the relative influence of drives on the net land sink has changed over the past century. There are fifteen models in the Version 1.0 release of MsTMIP simulation output^41^. Only those models that submitted all sensitivity simulations^18^ (SGw-SG3 for C-only models and SG1-BG1 for CN models) were included in this analysis.

**Table 1:** Semi-factorial design of MsTMIP simulations. For more details refer to Huntzinger *et al*.^18^.

Environmental Driver	Simulation				
	RG1	SG1	SG2	SG3	BG1
Climate	Constant	Time-varying	Time-varying	Time-varying	Time-varying
Land-cover change LCC		Constant			
Atmospheric CO_2_			Constant		
Nitrogen deposition				Constant	

### Mass-balance constraint

A global mass-balance estimate of net land sink strength, along with its associated uncertainty, was obtained from the Global Carbon Project (GCP)^2^ for 1959 to 2010. The net sink from the GCP is estimated by subtracting ocean uptake from fossil fuel emissions and the growth rate of atmospheric CO_2_. Thus, the GCP estimate provides a constraint on net uptake that takes into account all drivers of the terrestrial biosphere exchange, including land-use and land-cover change emissions^2^. Errors are calculated assuming that within each year the error on net uptake equals the root-sum-of-squares of ocean, atmospheric growth rate, and fossil fuel emissions. Errors on cumulative net uptake are assumed to correlated interannually (systematically biased) and therefore, additive in time.

### Calculation of β and γ (long-term sensitivity of global land carbon storage to CO_2_ and climate warming)

The response of the global net land sink to CO_2_ and climate (temperature and precipitation) was quantified using least-squares linear regression. To separate out the effects of CO_2_ (β) and climate warming (γ) on net uptake, we used the series of sensitivity simulations to isolate the impact of each driver. For both β and γ we used the full 110-yr simulation period spanning 1901 to 2010. Defining changes relative to 1901 provides the longest possible simulation period over which to diagnose sensitivities. The sensitivity of the net land sink from individual models to climate (γ) was determined by regressing the climate only (SG1) simulations^6^ against temperature and precipitation (obtained from the MsTMIP driver data). The regression coefficient and associated uncertainty (standard error) on temperature was used to diagnose γ. Other factors (not considered in the regression), such as solar radiation and wind speed (if there are any such trends in the weather driver data set), might also influence the trend in the net land sink. Therefore, the γ values can only be used for comparison across members of the ensemble rather than as an absolute measure of the climate sensitivity of individual models. To isolate the impact of CO_2_ concentrations on trends in the net land sink (β), the difference between the simulation accounting for time varying climate, land-cover change history, and atmospheric CO_2_ concentrations (SG3) and that containing only time-varying climate and land cover change (SG2) was regressed against time-varying CO_2_ concentrations from the MsTMIP driver data (refer to Huntzinger *et al*.^18^ (ref. 6) for more information about sensitivity simulations). Our use of linear regression in estimating β and γ does not account for any non-linearity in the sensitivity of net uptake to CO_2_ and temperature.

In both regressions, similar to Cox *et al*.^31^, we excluded the years 1963, 1964, 1982, 1983, 1991, and 1992, which were heavily influenced by volcanic eruptions. Volcanic events can affect land sink strength through changes in diffuse radiation, which may or may not be considered by the models in the ensembles but would likely influence the mass-balance estimate as a constraint. Therefore, to ensure consistency and comparability across models and with the mass balance constraint, these post volcano years were removed prior to the regression analysis.

We define β and γ as the sensitivity of net uptake to direct CO_2_ and climate (temperature) relative to 1901. The regression analysis retrieves sensitivities (coefficients) with units PgC/yr/ppm (or PgC/yr/K). Thus, each coefficient was multiplied the length of the reference time period (i.e., 104 which excludes volcano years) to remove the time dimension from the sensitivities and provide values more comparable to what are seen for coupled-carbon climate models.

### Calculation of γ_IAV_ (short-term sensitivity of global land carbon storage to climate warming) and observation-constrained γ

Least squares linear regression was also used to diagnose γ_IAV_, the short-term sensitivity of net carbon uptake to interannual variability (IAV) in temperature (as a representative for climate), for each model and the GCP observational constraint. These short-term sensitivities of net land sink strength to temperature are calculated over the period 1959 to 2010, the time period consistent with the GCP mass balance constraint. For the models, net uptake from the simulation SG3 (time-varying climate, land-cover change history and atmospheric CO_2_) or BG1 (time-varying climate, land-cover change history, atmospheric CO_2_, and nitrogen deposition) depending on whether the model includes carbon-nitrogen coupling. SG3/BG1 represents each model’s best estimate of net land uptake with all time-varying drivers turned on in the model (Refer to Huntzinger *et al*.^18^ (ref. 6) for more information about sensitivity simulations). This is the estimate most consistent with the mass-balance constraint. In order to isolate γ_IAV_, both the model estimates of the net land sink and annual global mean temperature were detrended by subtracting their 11-year running means. As with the calculation of β and γ, post-volcano years were removed prior to the regression.

Similar to previous studies^31, 32^, we find a tight correlation (R^2^ = 0.88; P < 10^−5^; Fig. 3b) between the short-term sensitivity of net carbon uptake to interannual variations in temperature (γ_IAV_) and the long-term sensitivity of global land carbon storage to climate warming (γ) (Fig. 3b and Figure S3). Interestingly, carbon-nitrogen models fall on the same linear line as carbon-only models (Fig. 3b), suggesting that the inclusion of nitrogen cycling does not alter the relationship between short- and long-term responses of land carbon uptake to climate. With the MsTMIP-derived relationship between γ and γ_IAV_ (linear relationship in Fig. 3b) and the GCP constraint on γ_IAV_, we identify an observational-constraint on γ (vertical grey shaded region in Fig. 3b).

## Electronic supplementary material

Supplemental Information
