# Supplementary material for: Uncertainty in the response of terrestrial carbon sink to environmental drivers undermines carbon-climate feedback predictions
Source: Sci Rep. 2017 Jul 6;7:4765. doi: 10.1038/s41598-017-03818-2 (PMC5500546; doi:10.1038/s41598-017-03818-2)
Supplement: Supplementary file 1 — Supplemental Information [file 41598_2017_3818_MOESM1_ESM.pdf]

Supplemental for:

Uncertainty in the response of terrestrial carbon sink to environmental drivers undermines carbon-climate feedback predictions

Huntzinger, D.N.[1*], A. M. Michalak[2], C. Schwalm[1,3], P. Ciais[4], A.W. King[5], Y. Fang[2], K. Schaefer[6], Y. Wei[5], R.B. Cook[5], J.B. Fisher[7], D. Hayes[8], M. Huang[9], A. Ito[10], A. K. Jain[11], H. Lei[12,13], C. Lu[14], F. Maignan[4], J. Mao[5], N. Parazoo[7], S. Peng[4], B. Poulter[15], D. Ricciuto[5], X. Shi[5], H. Tian[16], W. Wang[17], N. Zeng[18], F. Zhao[18]

[1] School of Earth Sciences and Environmental Sustainability, Northern Arizona University, P.O. Box 5694, Flagstaff, Arizona 86011-5694;
[2] Department of Global Ecology, Carnegie Institution for Science, Stanford, California
[3] Woods Hole Research Center, Falmouth MA 02540, USA
[4] Laboratoire des Sciences du Climat et de l'Environnement, IPSL-LSCE CEA CNRS UVSQ, 91191 Gif sur Yvette, France
[5] Environmental Sciences Division and Climate Change Science Institute, Oak Ridge National Laboratory, Oak Ridge, TN 37831, USA
[6] National Snow and Ice Data Center, Cooperative Institute for Research in Environmental Sciences, University of Colorado, Boulder, Colorado
[7] Jet Propulsion Laboratory, California Institute of Technology, Pasadena, CA USA
[8] School of Forest Resources, University of Maine, Orno, ME USA
[9] Atmospheric and Global Change Division, Pacific Northwest National Laboratory, Richland, WA USA
[10] National Institute for Environmental Studies, Tsukuba, Japan
[11] Department of Atmospheric Sciences, University of Illinois at Urbana-Champaign, Urbana, IL, USA
[12] Atmospheric Sciences and Global Change Division, Pacific Northwest National Laboratory, Richland, Washington
[13] State Key Laboratory of Hydroscience and Engineering, Department of Hydraulic Engineering, Tsinghua University, Beijing, China
[14] Department of Ecology, Evolution and Organismal Biology, Iowa State University, Ames, IA USA
[15] Department of Ecology, Montana State University, Bozeman, MT USA
[16] International Center for Climate and Global Change Research and School of Forestry and Wildlife Sciences, Auburn University, Auburn, Alabama, USA
[17] Ames Research Center, National Aeronautics and Space Administration, Moffett Field, California
[18] Department of Atmospheric and Oceanic Science, University of Maryland, College Park, Maryland

* Corresponding author: (Tel: +1-928-523-1669, Fax: +1-928-523-7423, deborah.huntzinger@nau.edu)

Supplemental for:
Uncertainty in the response of terrestrial carbon sink to environmental drivers undermines carbon-climate feedback predictions

Supplemental Information

Table S1. Cumulative net contribution in PgC of drivers to simulated global net land sink over the last 5 decades (1959-2010), along with the percent contribution of each driver to the total change (+ and -) in cumulative flux for that region. Attribution is done through simulation differencing. Thus, only models with that submitted simulations RG1 through BG1 (models with N-cycle) and RG1 through SG3 (models without N-cycle) are included. Models with a coupled carbon-nitrogen cycle are denoted in bold.

	Cumulative contribution (PgC)				Percent contribution to total change (%)			
	Climate	LCC	CO_2	N-dep	Climate	LCC	CO_2	N-dep
**CLM4**	6.13	-81.38	32.16	24.82	4.2%	56.3%	22.3%	17.2%
**CLM4ViC**	1.45	-56.97	30.20	22.93	1.3%	51.1%	27.1%	20.6%
**DLEM**	5.22	-0.08	42.33	24.63	7.2%	0.1%	58.6%	34.1%
**ISAM**	-26.81	-72.54	82.35	0.22	14.7%	39.9%	45.3%	0.1%
**TEM6**	3.34	23.28	75.18	19.45	2.8%	19.2%	62.0%	16.0%
GTEC	-25.78	-29.31	179.27		11.0%	12.5%	76.5%	
LPJ-wsl	-42.48	-64.58	111.38		19.4%	29.6%	51.0%	
ORCHIDEE-LSCE	-2.99	-18.87	103.18		2.4%	15.1%	82.5%	
SiB3	-12.08	-8.43	14.68		34.3%	24.0%	41.7%	
SiBCASA	-29.09	-43.98	292.47		8.0%	12.0%	80.0%	
VEGAS2.1	58.18	-35.42	24.54		49.2%	30.0%	20.8%	
VISIT	-10.99	52.28	140.87		5.4%	25.6%	69.0%	
Full model ensemble	-6.33 (25.75)	-28.00 (40.05)	94.05 (80.57)	18.41 (10.39)	13.3% (14.7%)	26.3% (16.5%)	53.1% (22.1%)	17.6% (12.1%)
Models with N-cycle	-2.13 (13.91)	-37.54 (46.44)	52.44 (24.59)	18.41 (10.39)	6.1% (5.3%)	33.3% (23.4%)	43.0% (18.0%)	17.6% (12.1%)
Models without N-cycle	-9.32 (32.58)	-21.19 (37.05)	123.77 (95.01)		18.5% (17.3%)	21.2% (7.9%)	60.2% (23.1%)	

Supplemental for:
Uncertainty in the response of terrestrial carbon sink to environmental drivers undermines carbon-climate feedback predictions

Table S2. Average land uptake and cumulative land uptake simulated by the MsTMIP models compared with the observation-based estimate from Global Carbon Project[1] and average uptake simulated by 11 coupled carbon-climate Earth System Models (ESM)[2]. Models that are in bold font include a dynamic nitrogen cycle. Also shown are the multi-model mean and range (1σ).

	Net C uptake ($PgC\ yr^{-1}$)			Cumulative C uptake (Pg C)	
	1960-1969	1990-1999	2000-2009	1959-2010	1901-2010
**CLM4**	-1.22	-0.55	1.07	-18.27	-70.29
**CLM4VIC**	-0.93	-0.12	1.10	2.38	-36.16
**DLEM**	0.78	1.42	1.95	72.10	102.59
**ISAM**	-0.70	-0.41	0.06	-16.77	-56.62
**TEM6**	2.19	2.10	3.03	121.25	158.72
GTEC	1.12	2.53	3.77	124.18	160.83
LPJ-wsl	-1.34	0.23	1.90	4.33	-71.08
ORCHIDEE-LSCE	0.69	1.88	2.26	81.33	101.97
SIB3-JPL	-0.17	-0.06	-0.20	-5.83	-29.29
SIB-CASA	2.62	5.05	5.82	219.4	341.99
VEGAS2.1	0.09	0.99	1.60	47.30	58.18
VISIT	12.88	3.06	5.08	182.16	276.44
Full model ensemble	0.50 ± 1.5	1.34 ± 1.7	2.29 ± 1.9	67.4 ± 80.7	78.1 ± 138.5
Models with N-cycle	0.02 ± 1.4	0.49 ± 1.2	1.44 ± 1.1	31.2 ± 62.6	19.6 ± 104.1
Models without N-cycle	0.84 ± 1.5	1.95 ± 1.8	2.89 ± 2.1	93.3 ± 86.4	119.9 ± 151.9
Observation-based estimate (GCP)[1]	0.23 ± 0.8	1.06 ± 0.8	1.46 ± 0.8	34.6 ± 41.6	
CMIP5 models[2]		0.8 ± 0.7			

Supplemental for:
Uncertainty in the response of terrestrial carbon sink to environmental drivers undermines carbon-climate feedback predictions

Table S3. Cumulative net contribution in PgC of drivers to simulated net land sink over the last 5 decades (1959-2010) for the tropics (30S to 30N), along with the percent contribution of each driver to the total change (+ and -) in cumulative flux for that region. Attribution is done through simulation differencing. Thus, only models with that submitted simulations RG1 through BG1 (models with N-cycle) and RG1 through SG3 (models without N-cycle) are included. Models with a coupled carbon-nitrogen cycle are denoted in bold.

	Cumulative contribution (PgC)				Percent contribution to total change (%)			
	Climate	LCC	CO_2	N-dep	Climate	LCC	CO_2	N-dep
**CLM4**	-0.85	-64.89	22.34	13.62	0.8%	63.8%	22.0%	13.4%
**CLM4ViC**	-2.49	-48.05	21.67	12.94	2.9%	56.4%	25.5%	15.2%
**DLEM**	-0.07	-4.63	29.12	13.75	0.2%	9.7%	61.2%	28.9%
**ISAM**	-12.05	-61.50	53.50	0.04	9.5%	48.4%	42.1%	0.0%
**TEM6**	-8.64	9.15	44.04	8.34	12.3%	13.0%	62.8%	11.9%
GTEC	-31.90	-11.62	122.71		19.2%	7.0%	73.8%	
LPJ-wsl	-46.70	-40.74	58.98		31.9%	27.8%	40.3%	
ORCHIDEE-LSCE	-12.21	-2.24	54.49		17.7%	3.2%	79.0%	
SiB3	-5.50	-7.00	11.98		22.5%	28.6%	48.9%	
SiBCASA	-29.35	-25.76	233.38		10.2%	8.9%	80.9%	
VEGAS2.1	10.59	-25.22	16.41		20.3%	48.3%	31.4%	
VISIT	-32.09	46.40	96.76		18.3%	26.5%	55.2%	
Full model ensemble	-14.27 (16.96)	-19.68 (31.71)	63.78 (62.92)	9.74 (5.86)	13.8% (9.6%)	28.5% (21.1%)	51.9% (20.3%)	13.9% (10.3%)
Models with N-cycle	-4.82 (5.26)	-33.99 (34.03)	34.13 (14.08)	9.74 (5.86)	5.1% (5.4%)	38.3% (25.2%)	42.7% (19.2%)	13.9% (10.3%)
Models without N-cycle	-21.02 (19.52)	-9.46 (27.91)	84.96 (76.62)		20.0% (6.5%)	21.5% (16.0%)	58.5% (19.7%)	

Supplemental for:
Uncertainty in the response of terrestrial carbon sink to environmental drivers undermines carbon-climate feedback predictions

Table S4. Cumulative net contribution in PgC of drivers to simulated net land sink over the last 5 decades (1959-2010) for the northern mid-latitudes (>30N and <50N), along with the percent contribution of each driver to the total change (+ and -) in cumulative flux for that region. Attribution is done through simulation differencing. Thus, only models with that submitted simulations RG1 through BG1 (models with N-cycle) and RG1 through SG3 (models without N-cycle) are included. Models with a coupled carbon-nitrogen cycle are denoted in bold.

	Cumulative contribution (PgC)				Percent contribution to total change (%)			
	Climate	LCC	CO_2	N-dep	Climate	LCC	CO_2	N-dep
**CLM4**	5.74	-10.80	5.00	8.46	19.1%	36.0%	16.7%	28.2%
**CLM4ViC**	3.37	-5.53	4.24	7.54	16.3%	26.7%	20.5%	36.4%
**DLEM**	1.97	4.06	5.89	7.89	9.9%	20.5%	29.7%	39.8%
**ISAM**	-3.41	-8.41	11.16	0.09	14.8%	36.5%	48.4%	0.4%
**TEM6**	4.98	13.57	13.70	8.36	12.3%	33.4%	33.7%	20.6%
GTEC	0.54	-6.71	29.54		1.5%	18.2%	80.3%	
LPJ-wsl	4.93	-15.15	18.41		12.8%	39.4%	47.8%	
ORCHIDEE-LSCE	0.31	-8.99	21.78		1.0%	28.9%	70.1%	
SiB3	-2.67	-0.85	1.70		51.2%	16.2%	32.5%	
SiBCASA	1.84	-10.11	25.27		4.9%	27.2%	67.9%	
VEGAS2.1	28.16	-5.15	4.50		74.5%	13.6%	11.9%	
VISIT	10.41	2.36	20.12		31.6%	7.2%	61.2%	
Full model ensemble	4.68 (8.30)	-4.31 (7.90)	13.44 (9.41)	6.47 (3.58)	20.8% (21.9%)	25.3% (10.2%)	43.4% (22.7%)	25.1% (15.7%)
Models with N-cycle	2.53 (3.63)	-1.42 (10.11)	8.00 (4.18)	6.47 (3.58)	14.5% (3.6%)	30.6% (6.9%)	29.8% (12.4%)	25.1% (15.7%)
Models without N-cycle	6.21 (10.54)	-6.37 (5.86)	17.33 (10.41)		25.4% (28.5%)	21.5% (10.9%)	53.1% (24.0%)	

Supplemental for:
Uncertainty in the response of terrestrial carbon sink to environmental drivers undermines carbon-climate feedback predictions

Table S5. Cumulative net contribution in PgC of drivers to simulated net land sink over the last 5 decades (1959-2010) for the arctic-boreal region (>50N), along with the percent contribution of each driver to the total change (+ and -) in cumulative flux for that region. Attribution is done through simulation differencing. Thus, only models with that submitted simulations RG1 through BG1 (models with N-cycle) and RG1 through SG3 (models without N-cycle) are included. Models with a coupled carbon-nitrogen cycle are denoted in bold.

	Cumulative contribution (PgC)				Percent contribution to total change (%)			
	Climate	LCC	CO_2	N-dep	Climate	LCC	CO_2	N-dep
**CLM4**	1.45	-4.06	4.23	2.01	12.4%	34.6%	36.0%	17.1%
**CLM4ViC**	0.86	-2.04	3.73	1.83	10.2%	24.2%	44.1%	21.6%
**DLEM**	3.08	1.58	5.99	2.05	24.2%	12.5%	47.2%	16.2%
**ISAM**	-10.66	-0.64	16.07	0.10	38.8%	2.3%	58.5%	0.3%
**TEM6**	5.66	-7.26	14.43	2.02	19.3%	24.7%	49.1%	6.9%
GTEC	4.75	-5.46	20.76		15.4%	17.6%	67.0%	
LPJ-wsl	0.41	-6.10	31.78		1.1%	15.9%	83.0%	
ORCHIDEE-LSCE	8.53	-3.38	22.65		24.7%	9.8%	65.5%	
SiB3	-3.96	0.11	0.07		95.6%	2.6%	1.8%	
SiBCASA	-3.42	-2.67	29.05		9.7%	7.6%	82.7%	
VEGAS2.1	15.65	-2.00	3.07		75.5%	9.6%	14.8%	
VISIT	5.46	0.73	19.25		21.5%	2.9%	75.7%	
Full model ensemble	2.32 (6.68)	-2.60 (2.79)	14.26 (10.76)	1.60 (0.85)	29.0% (28.4%)	13.7% (10.1%)	52.1% (25.5%)	12.4% (8.6%)
Models with N-cycle	0.08 (6.28)	-2.48 (3.37)	8.89 (5.90)	1.60 (0.85)	21.0% (11.4%)	19.6% (12.5%)	47.0% (8.2%)	12.4% (8.6%)
Models without N-cycle	3.92 (6.96)	-2.68 (2.57)	18.09 (12.16)		34.8% (36.0%)	9.4% (5.8%)	55.8% (33.4%)	

Supplemental for:
Uncertainty in the response of terrestrial carbon sink to environmental drivers undermines carbon-climate feedback predictions

Table S6. Percent of global land area were each driver is the dominant factor controlling the trend in land sink from 1959 to 2010. Only models that submitted simulations RG1 through BG1 (models with N-cycle) and RG1 through SG3 (models without N-cycle) are included. Models with a coupled carbon-nitrogen cycle are denoted in bold.

	Decrease in sink				Increase in sink (%)			
	Climate	LCC	CO_2	N-dep	Climate	LCC	CO_2	N-dep
**CLM4**	20.6%	20.7%	0.43%	0.43%	14.5%	6.1%	15.9%	21.4%
**CLM4ViC**	19.0%	24.2%	0.25%	0.39%	11.1%	10.6%	13.5%	21.0%
**DLEM**	22.6%	7.6%	0.04%	0.01%	7.1%	9.4%	37.5%	15.7%
**ISAM**	23.0%	22.7%	0.00%	0.00%	3.6%	3.1%	47.5%	0.0%
**TEM6**	16.1%	17.7%	0.07%	0.05%	6.6%	38.6%	18.0%	2.9%
GTEC	21.2%	11.7%	0.15%		8.4%	3.4%	55.0%	
LPJ-wsl	23.4%	17.9%	1.05%		15.5%	3.8%	38.3%	
ORCHIDEE-LSCE	19.2%	13.9%	0.19%		9.5%	8.0%	49.2%	
SiB3	16.6%	3.6%	0.92%		49.3%	10.1%	19.4%	
SiBCASA	7.7%	5.2%	0.06%		5.5%	4.4%	77.1%	
VEGAS2.1	22.8%	20.3%	0.00%		43.0%	2.5%	11.4%	
VISIT	22.1%	0.1%	0.60%		33.5%	10.4%	33.3%	
Full model ensemble	19.5% (4.3%)	13.8% (7.7%)	0.3% (0.3%)	0.2% (0.2%)	17.3% (14.9%)	9.2% (9.3%)	34.7% (19.3%)	12.2% (9.1%)
Models with N-cycle	20.2% (2.8%)	18.6% (6.6%)	0.16% (0.18%)	0.18% (0.22%)	8.6% (4.2%)	13.6% (14.3%)	26.5% (15.1%)	12.2% (10.1%)
Models without N-cycle	19.0% (5.5%)	10.4% (7.6%)	0.42% (0.43%)		23.5% (18.1%)	6.1% (3.3%)	40.5% (22.3%)	

Supplemental for:
Uncertainty in the response of terrestrial carbon sink to environmental drivers undermines carbon-climate feedback predictions

Table S7. Sensitivity of the global net land sink to atmospheric CO_2 (β), temperature (γ), and temperature variability (γ_{IAV}) for each model and for a mass balance observational product based on the Global Carbon Project (GCP) estimate of the net land sink. Sensitivities are calculated from 1901 to 2010 (β and γ) and 1959 to 2010 (γ_{IAV}), excluding post volcano years (1963, 1964, 1982, 1983, 1991). Table also shows the associated carbon gain/loss since 1901 as a result of these sensitivities, as well as the relative contribution of each. Values in parentheses next to the sensitivities for each model and for the observational product (GCP) indicate the uncertainties (standard error) of each coefficient (β, γ, γ_{IAV}) based on the regression. The values in parentheses next to the ensemble mean values of each coefficient indicate the standard deviation across model sensitivities.

	Global			C gain/loss since 1901 due to:		Relative impact
	β (PgC ppm^{-1})	γ (PgC K^{-1})	γ_{IAV} (PgC yr^{-1} K^{-1})	CO_2 (PgC)	Temperature (PgC)	Temp / CO_2
**CLM4**	0.91 (0.02)	-25.81 (17.39)	-0.94 (0.47)	85.12	-28.39	0.33
**CLM4ViC**	0.84 (0.02)	-23.37 (11.61)	-0.69(0.50)	78.57	-25.71	0.33
**DLEM**	1.33 (0.05)	-131.16 (19.12)	-2.09 (0.88)	124.41	-144.28	1.16
**ISAM**	2.43 (0.04)	-175.61 (13.05)	-2.86 (0.49)	227.30	-193.17	0.85
**TEM6**	2.15 (0.12)	-94.97 (21.74)	-1.58 (0.65)	201.11	-104.47	0.52
GTEC	4.93 (0.11)	-259.65 (27.28)	-3.62 (1.11)	461.15	-285.62	0.62
LPJ-wsl	3.35 (0.10)	-191.75 (25.03)	-3.60 (0.65)	313.36	-210.93	0.67
ORCHIDEE-LSCE	2.06 (0.08)	-109.31 (15.81)	-2.30 (0.62)	192.69	-120.24	0.62
SiB3	0.28 (0.42)	-54.44 (18.60)	-0.63 (0.60)	26.19	-59.88	2.29
SiBCASA	8.21 (0.08)	-112.70 (12.13)	-1.30 (0.54)	767.96	-123.97	0.16
VEGAS2.1	0.69 (0.02)	-64.2 (18.43)	-1.57 (0.80)	64.54	-70.62	1.09
VISIT	4.13 (0.06)	-154.29 (20.67)	-2.70 (0.83)	386.32	-169.72	0.44
Full model ensemble	2.61 (2.3)	-116.44 (70.9)	-1.99 (1.1)	244.1 (212.8)	-128.1 (78.0)	0.76 (0.6)
Models with N-cycle	1.53 (0.7)	-90.01 (66.6)	-1.63 (0.9)	143.3 (67.7)	-99.2 (73.0)	0.64 (0.4)
Models without N-cycle	3.38 (2.7)	-135.19 (72.8)	-2.25(1.1)	316.0 (255.8)	-148.7 (80.1)	0.84 (0.7)
Obs (GCP)			-2.24 (0.8)			

Supplemental for:
Uncertainty in the response of terrestrial carbon sink to environmental drivers undermines carbon-climate feedback predictions

Table S8. The MsTMIP environmental driver data summary. Adapted from Wei et al. 2014. All driver data sets a global and processed to 0.5° by 0.5° spatial resolution.

Category	Name	Native Temporal Period, Resolution	Extended Temporal Period, Resolution[a]	Variables
**Climate**	CRU-NCEP[b]	1901-2010, 6-hourly	1801-2010, 6-hourly	– precipitation<br>– air temperature<br>– air specific humidity<br>– air relative humidity (NA only)<br>– pressure<br>– downward longwave radiation downward shortwave radiation<br>– wind speed
**Land Water Mask**	CRU-NCEP	constant	constant	binary land vs. water map
**CO_2**	Extended GLOBALVIEW-CO_2	1801-2010, monthly	1801-2010, monthly	atmospheric CO_2 concentration
**Nitrogen Deposition**	Enhanced Dentener	1860-2010, annual	1801-2010, annual	NHx-N deposition<br>NOy-N deposition
**Land Cover Change**	SYNMAP+ Hurtt	1801-2010, annual	1801-2010, annual	land cover state maps
**C3/C4 Grass**	C3/C4 grass fraction	constant	constant	relative fractions of C3/C4 grasses
**Major Crops**	Monfreda et al. 2008	constant	constant	fraction of harvest area in each grid cell for maize, rice, soybean, and wheat
**Phenology**	GIMMSg	1801-2010, monthly	1801-2010, monthly	NDVI, LAI, and fPAR
**Soil**	HWSD v1.1	constant	constant	– soil layers<br>– dominant soil type<br>– reference soil depth<br>– clay/sand/silt fractions<br>– pH<br>– organic carbon<br>– cation exchange capacity<br>– reference bulk density<br>– gravel content

[a] Native temporal periods of environmental driver data sets compiled for MsTMIP are extended to be compatible with the simulation time period (1801-2010) defined by MsTMIP.

[b] **CRU-NCEP**: Climate Research Unit, National Centers for Environmental Prediction; **SYNMAP**: SYNergetic land cover MAP; **GIMMSg**: Global Inventory Monitoring and Modeling System version g; **NDVI**: Normalized Difference Vegetation Index; **LAI**: Leaf Area Index; **fPAR**: fraction of Photosynthetically Active Radiation; **HWSD**: Harmonized World Soil Database.

Supplemental for:
Uncertainty in the response of terrestrial carbon sink to environmental drivers undermines carbon-climate feedback predictions

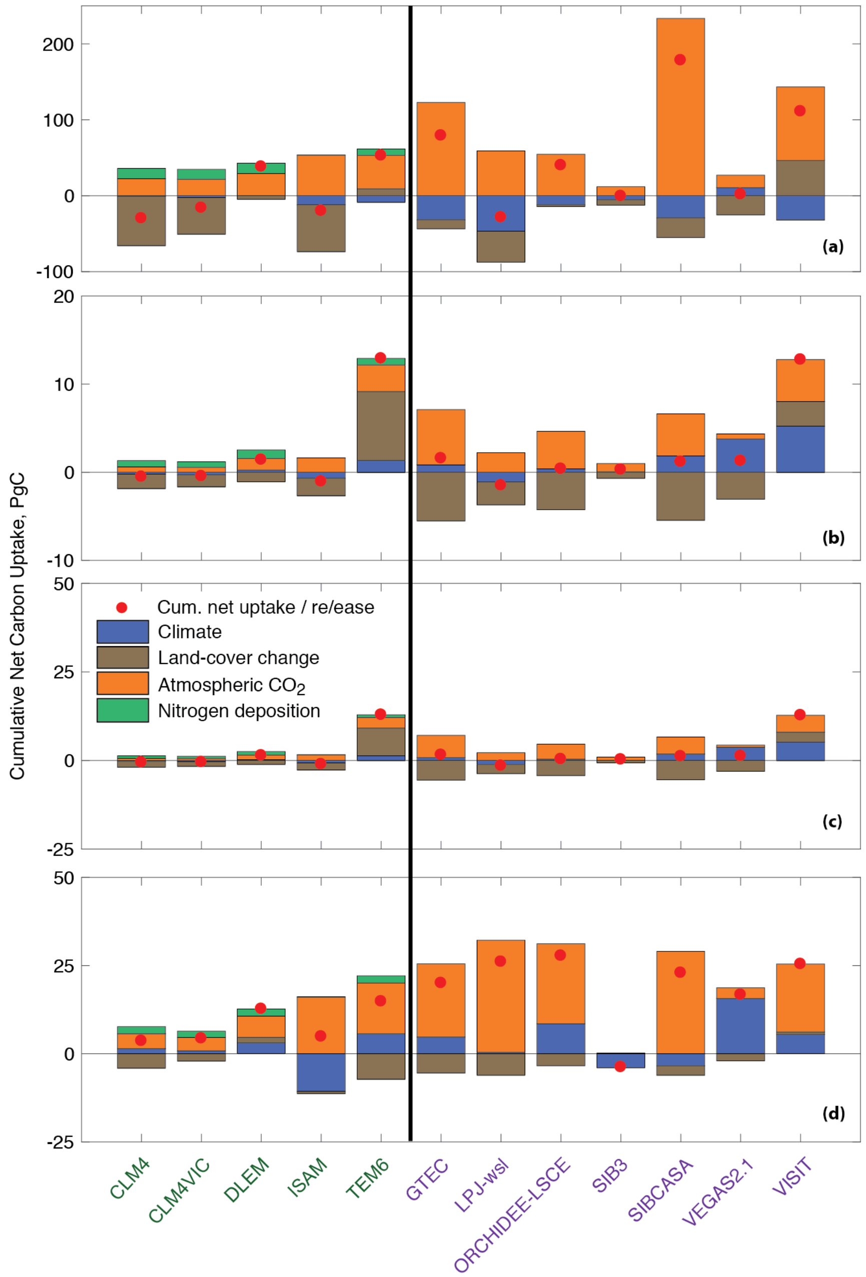

Figure S1. Attribution of the cumulative net sink (red circles) since 1959 decomposed by driver and by model for (a) the tropics; (b) extratropics; (c) northern midlatitudes; and (d) arctic-boreal regions, Drivers include: climate (blue), land-cover change (brown), atmospheric CO_2 (orange), and nitrogen deposition (green). Models are grouped based on whether they include (left; purple) or do not include (right; green) a coupled carbon-nitrogen cycle. (Figure was created using Matlab version R2015a (http://www.mathworks.com/products/matlab/) with post processing done in Adobe Illustrator CS6 Version 16.04 (https://www.adobe.com/products/illustrator.html).

Supplemental for:
Uncertainty in the response of terrestrial carbon sink to environmental drivers undermines carbon-climate feedback predictions

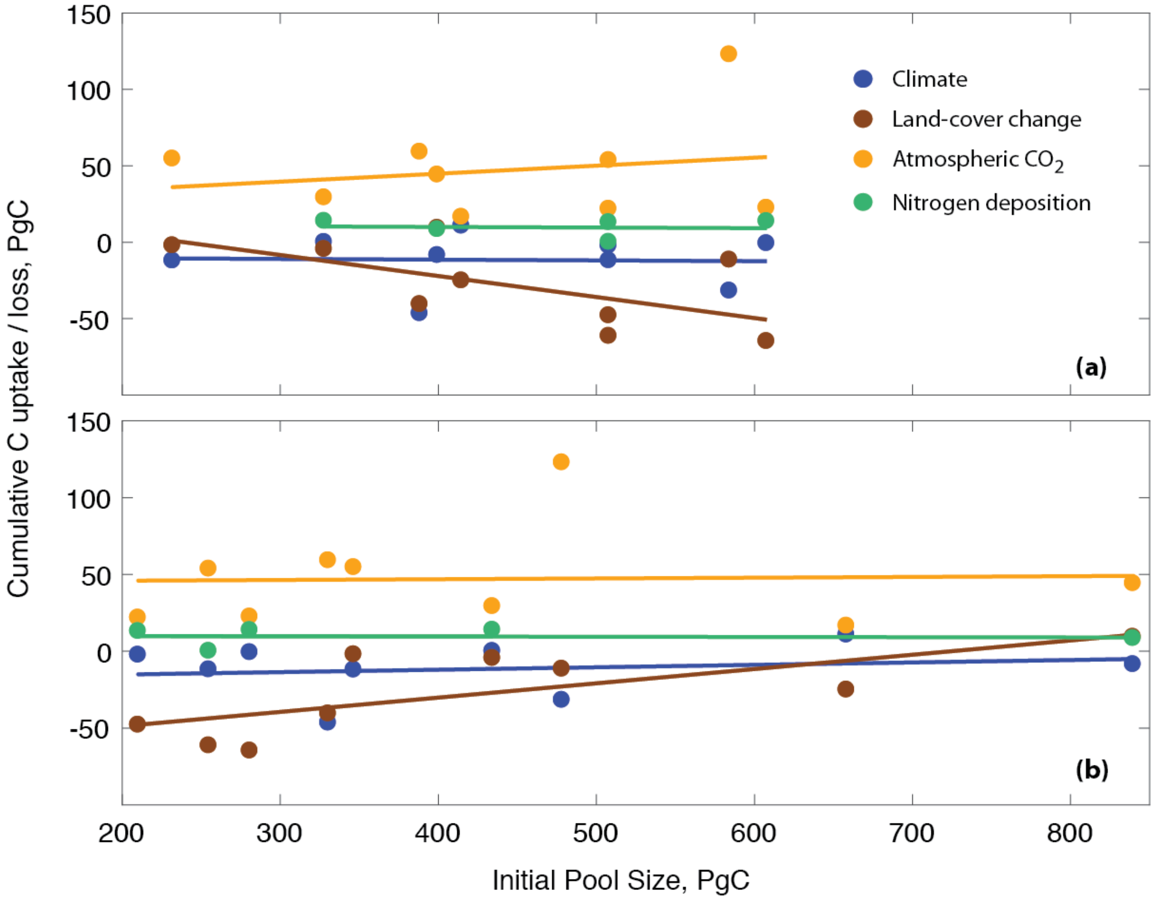

Figure S2. Steady-state tropical (a) live biomass pool size and (b) soil carbon pool size against cumulative land carbon uptake / loss in the tropics since 1959 attributed to driver. Circles show individual models (n=9); lines show trend across models. Models not included: Biome-BGC, SiB3.0, SiB-CASA, and VISIT. Biome-BGC did not submit sensitivity simulations for land-cover change and atmospheric CO_2; SiB3.0 does not have carbon pools; VISIT did not provide stead-state soil carbon pools; and SiB-CASA reported very large total living biomass (>1,600 PgC) which skewed the best-fit line in (a).

Supplemental for:
Uncertainty in the response of terrestrial carbon sink to environmental drivers undermines carbon-climate feedback predictions

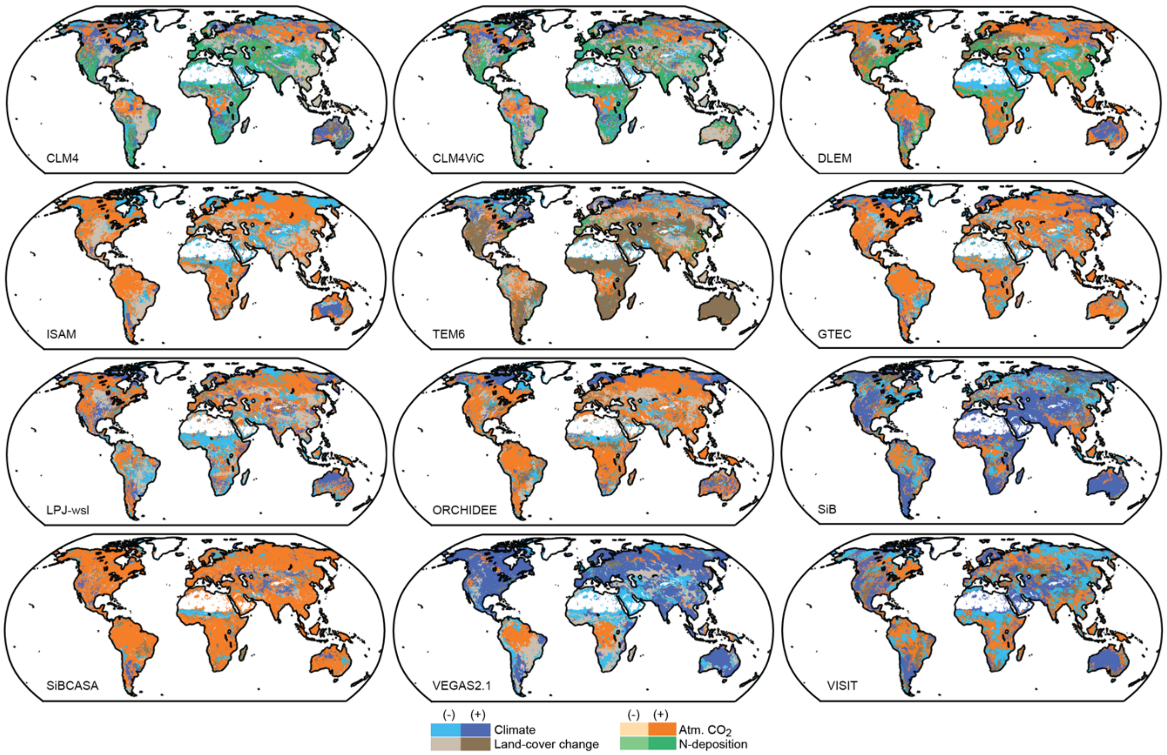

Figure S3. Dominate driver of the cumulative net land sink over the simulation period 1959 to 2010 for each model, and whether that driver is associated with a enhancement (+) or weakening (-) of land carbon sink strength over that time period. Drivers include: climate (blue), land-cover change (brown), atmospheric CO_2 (orange), and nitrogen deposition (green). Figure was created using Matlab version R2015a (http://www.mathworks.com/products/matlab/) with post processing done in Adobe Illustrator CS6 Version 16.04 (https://www.adobe.com/products/illustrator.html).

Supplemental for:
Uncertainty in the response of terrestrial carbon sink to environmental drivers undermines carbon-climate feedback predictions

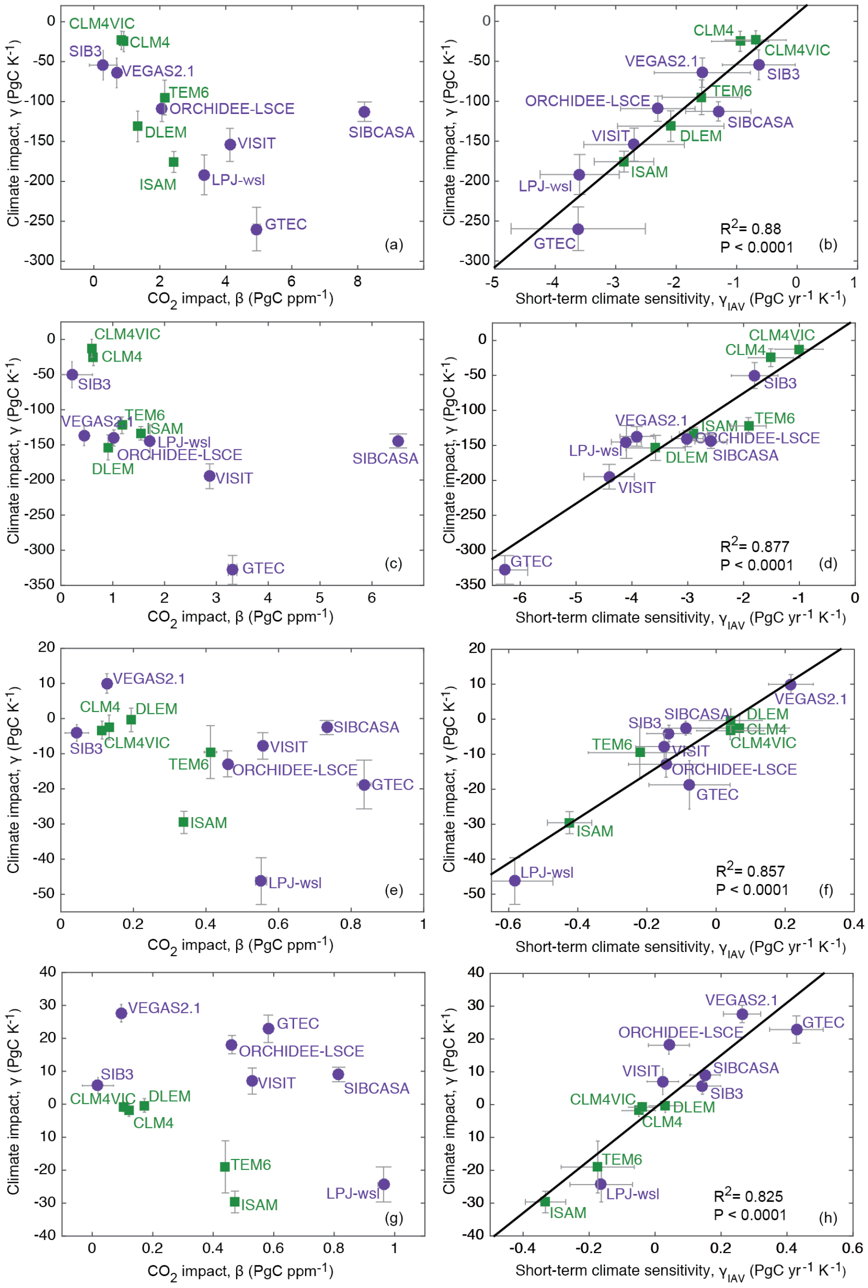

Figure S4. Impact of climate and atmospheric CO_2 on (a,b) global, (c,d) tropical, (e,f) northern mid-latitude, and (g,h) arctic-boreal land carbon uptake. (a,c,e,f) The response of the net land sink over the period 1901 to 2010 to rising atmospheric CO_2 (β) and temperature (γ) estimated from multi-linear regression for models with (green) and without (purple) a dynamic nitrogen cycle. (b,d,f,h) The long-term sensitivity of the net land sink to climate warming (γ) versus the short-term sensitivity of net uptake to interannual variability in temperature (γ_{IAV}). The error bars show uncertainty (s.e.) in the regression coefficients (β, γ, γ_{IAV}). The black lines show the best-fit, linear relationship between short- and long-term climate sensitivity. Figure was created using Matlab version R2015a

Supplemental for:
Uncertainty in the response of terrestrial carbon sink to environmental drivers undermines carbon-climate feedback predictions

(http://www.mathworks.com/products/matlab/) with post processing done in Adobe Illustrator CS6 Version 16.04 (https://www.adobe.com/products/illustrator.html).
